# Persistent SARS-CoV-2 Infection, EBV, HHV-6 and Other Factors May Contribute to Inflammation and Autoimmunity in Long COVID

**DOI:** 10.3390/v15020400

**Published:** 2023-01-31

**Authors:** Aristo Vojdani, Elroy Vojdani, Evan Saidara, Michael Maes

**Affiliations:** 1 Immunosciences Lab, Inc., Los Angeles, CA 90035, USA; 2 Cyrex Laboratories, LLC, Phoenix, AZ 85034, USA; 3 Regenera Medical, Los Angeles, CA 90025, USA; 4 Sackler Faculty of Medicine, Tel Aviv University, Ramat Aviv, Tel Aviv 69978, Israel; 5 Department of Psychiatry, Faculty of Medicine, Chulalongkorn University, 1873 Rama 4 Road, Pathumwan, Bangkok 10330, Thailand

**Keywords:** long COVID, autoimmunity, myalgic encephalomyelitis/chronic fatigue syndrome (ME/CFS), viral reactivation, EBV, HHV-6, gut microbiota

## Abstract

A novel syndrome called long-haul COVID or long COVID is increasingly recognized in a significant percentage of individuals within a few months after infection with SARS-CoV-2. This disorder is characterized by a wide range of persisting, returning or even new but related symptoms that involve different tissues and organs, including respiratory, cardiac, vascular, gastrointestinal, musculo-skeletal, neurological, endocrine and systemic. Some overlapping symptomatologies exist between long COVID and myalgic encephalomyelitis/chronic fatigue syndrome (ME/CFS). Very much like with long ME/CFS, infections with herpes family viruses, immune dysregulation, and the persistence of inflammation have been reported as the most common pattern for the development of long COVID. This review describes several factors and determinants of long COVID that have been proposed, elaborating mainly on viral persistence, reactivation of latent viruses such as Epstein–Barr virus and human herpesvirus 6 which are also associated with the pathology of ME/CFS, viral superantigen activation of the immune system, disturbance in the gut microbiome, and multiple tissue damage and autoimmunity. Based on these factors, we propose diagnostic strategies such as the measurement of IgG and IgM antibodies against SARS-CoV-2, EBV, HHV-6, viral superantigens, gut microbiota, and biomarkers of autoimmunity to better understand and manage this multi-factorial disorder that continues to affect millions of people in the world.

## 1. Introduction

Many individuals infected with SARS-CoV-2 do not show severe symptoms and completely recover from the virus after a short period of time. However, there are others that experience a range of symptoms including flu-like, respiratory, digestive and cognitive symptoms, loss of smell or taste, fatigue, and skin lesions. The severity and recovery time for each case varies depending on each patient’s previous health history [1,2]. This inter-individual difference in combatting the virus is dependent on the gene as well as the exposome [3,4]. The exposome is an individual’s lifetime exposure to a variety of external and internal factors [5,6].

Although most COVID-19 infections resolve within two weeks, prolonged follow-up case studies have shown that approximately 30% of individuals who have supposedly recovered from the disease continue to suffer from some sort of associated symptomatology over an extended period of time. This phenomenon has been termed many names, from Post-Acute COVID Syndrome (PACS) to Post-Acute Sequelae of COVID-19 (PASC) to long-haul COVID and many others, but it is most commonly known as long COVID [7,8,9].

The World Health Organization (WHO) describes long COVID as a wide range of new, returning or ongoing health problems that people experience more than a month after initial infection with the SARS-CoV-2 virus. The main symptoms of long COVID currently include fatigue, brain fog, difficulty sleeping, arthralgia, myalgia, pharyngitis, fever, headaches, gastrointestinal symptoms, skin rashes, and affective symptoms including depression and anxiety, which are symptoms similar to those that present during the initial infection [10,11,12,13,14,15].

Patients who suffer from these symptoms had different degrees of COVID-19 severity [9]. Overall, though, the more severe degrees of COVID-19 infection are associated with multiple early factors and the induction of more severe long COVID [15]. This is indicated by findings that a large part of the variance in long COVID fatigue and affective symptoms is predicted by increased peak body temperature and lowered blood oxygenation, which both indicate activate immune-inflammatory pathways, during acute COVID-19 infection [15].

Although the causes of long COVID are beginning to emerge, understanding the complex biology and pathophysiology of this disorder is a crucial step toward the prevention, treatment, or reversal of this condition [16]. In this regard, we may learn from the role of various pathogens in myalgic encephalomyelitis/chronic fatigue syndrome (ME/CFS), which is one of the major symptoms of long COVID [17]. ME/CFS is a complicated chronic illness in which fatigue and exhaustion reduce a person’s physical activity by more than 50%, leaving the individual bed-ridden or house-bound [18,19]. Very much like with long COVID, infections have been reported as the most common pattern for the onset of ME/CFS, especially when the beneficial sickness behavior responses are insufficient [20,21].

In fact, over the years, several viruses, including Epstein–Barr virus (EBV) and human herpes type 6 (HHV-6) have been studied as proposed models for the pathophysiology of ME/CFS [17,21,22,23,24,25,26]. These and other viral infections may be responsible for the overlapping symptomatologies such as fatigue, post-exertional malaise, cognitive dysfunction, sleep disturbance, affective symptoms and lightheadedness that are found in both long COVID and ME/CFS [27]. How these chronic symptoms develop is unclear, but in the case of SARS-CoV-2, persisting SARS-CoV-2 viremia has been established in different tissues that widely express angiotensin-converting enzyme 2 (ACE2) receptors [28]. The reactivation of dormant viruses such as EBV and HHV-6 due to immune dysregulation and the activation of the interferon cascade, induction of the NLRP3 inflammasome, increased oxidative damage and lowered antioxidant defenses (the glutathione system and zinc), infection, neuro-inflammation including the brainstem, molecular mimicry and autoimmunity, and, of course, underlying genetic predisposition have been hypothesized as potential triggers of long COVID [27,29,30,31].

**Figure 1:**
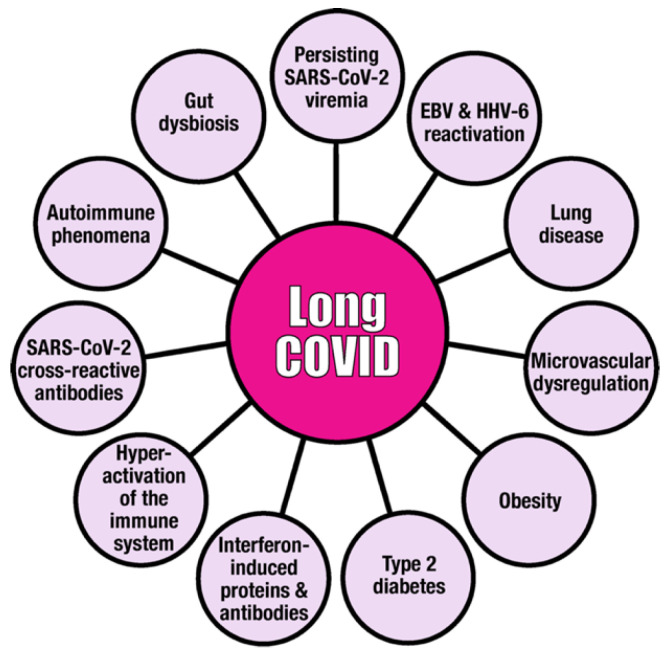
Major factors that may be involved in the pathophysiologic mechanism of long COVID disease. This figure illustrates that long COVID is a multi-factorial disease, and that SARS-CoV-2, EBV and HHV-6 are viral contributors to its development, progress, duration and/or severity.

There are other factors and certain comorbidities, such as pre-existing diabetes mellitus, obesity, lung disease and microvascular dysregulation, that contribute to long COVID. Major contributors are shown in Figure 1 and will be discussed in this manuscript.

**Figure 2:**
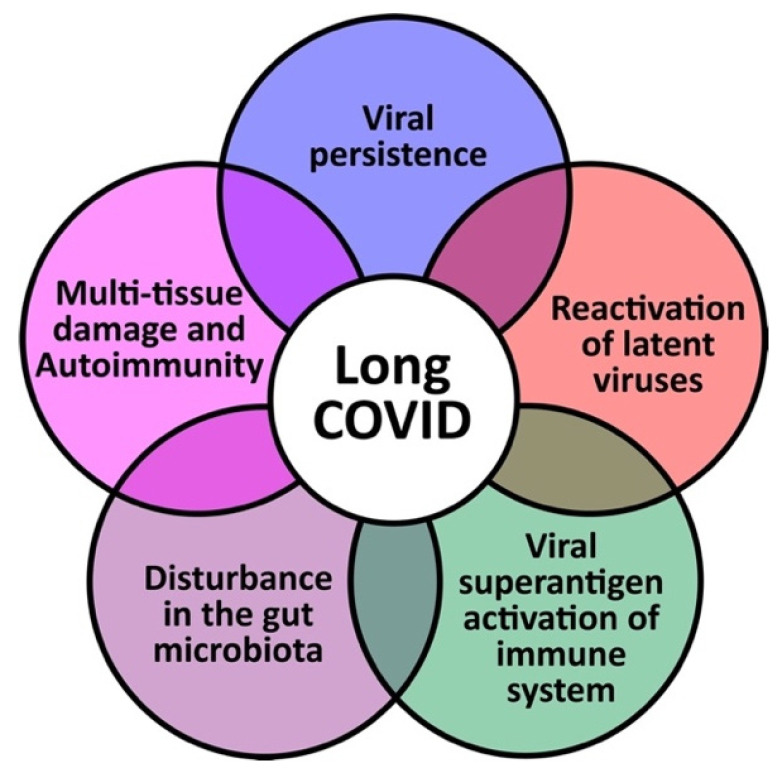
The 5 hypothesized mechanisms of long COVID disease.

Any potential discoveries regarding the pathophysiologic mechanisms for long COVID will increase opportunities for our understanding of the disease and the development of treatments for improved outcome [25]. Currently, it is hypothesized that long COVID could occur through five mechanisms: (1) Viral persistence; (2) Reactivation of latent viruses; (3) Viral superantigen activation of the immune system; (4) Disturbance in the gut microbiome; and (5) Multiple tissue damage and autoimmunity. These mechanisms are summarized in Figure 2 and discussed below.

There are other lesser-known factors that may also be associated with long COVID. One such is the impact of nitro-oxidative stress, which was studied by Al-Hakeim et al. [32]. The team examined the effects of peripheral oxygen saturation and peak body temperature on the immune, oxidative and nitrosative stress pathways and neuropsychiatric symptoms of long COVID. Their results suggest that post-viral somatic and mental symptoms of the disease may have a neuroimmune and neuro-oxidative origin [32].

## 2. Viral Persistence

### 2.1. Background: Mechanism for Viral Infection

First, how does the SARS-CoV-2 virus infect a host?

**Figure 3:**
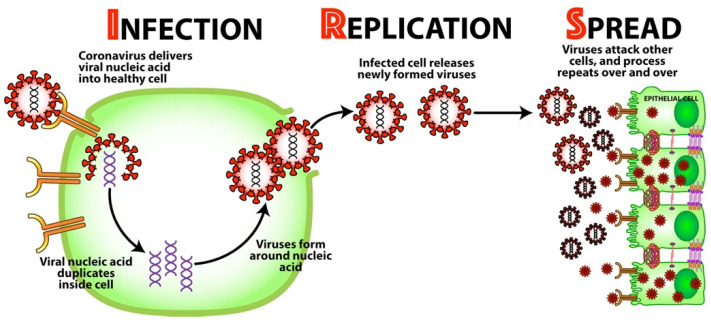
How SARS-CoV-2 spreads throughout the body: infection, replication, and spread.

Angiotensin-converting enzyme 2 or ACE2 receptor is the protein that provides the entry point for the SARS-CoV-2 virus to enter human cells. They are present in the respiratory system, vascular endothelium, heart, brain, GI tract, kidney, liver, spleen and pancreas [30]. After binding to the ACE2 receptor, SARS-CoV-2 uses three important steps to establish itself all over the body (see Figure 3):

- Infection—Once bound to the receptor, the virus delivers its viral nucleic acid into the healthy cell.
- Replication—The viral nucleic acid duplicates inside the cell, and the cell’s resources are used to form more viruses around the nucleic acid. The now-infected cell releases these newly formed viruses.
- Spread—The newly formed viruses now go on to attack other cells all throughout the body, and the process repeats over and over.

Depending on the strength of the host’s immune system, the spreading of this virus may induce mild symptoms or induce life-threatening disease [30].

### 2.2. What Is Viral Persistence?

Under certain conditions, a viral invader may not be completely eliminated by the host’s immune system. The cessation of symptoms, non-detection of viral presence, and the development of immunity after infection does not necessarily mean that the virus has been completely eradicated from the host [33]. Some viruses or their parts may remain and hide in tissues and can be reactivated later. This is viral persistence [34,35,36].

Some viral pathogens have the capability to avoid recognition and thus avoid activation of the proper immune response [36].

The viral RNA may survive elimination and hide in tissue reservoirs, waiting for reactivation, replication and spread [35].

The idea that viral persistence might be responsible for long COVID came from various studies from different parts of the world that found viral RNA or viral antigens in the blood, feces and different tissue biopsies from 3 to several months after the initial diagnosis of COVID-19 [37,38,39,40,41].

Persistent SARS-CoV-2 infection and superantigens (SAgs) released by the virus which are known to induce polyclonal activation of T cells and cytokine storm could also induce both apoptosis of cellular components and direct activation of dendritic cells, which could lead to the autoimmunity associated with long COVID [42]. Because the persistence of SARS-CoV-2 and its remnants in the tissue results in immune response to the virus, the measurement of IgG and IgM antibody against SARS-CoV-2 spike and nucleoprotein is recommended.

## 3. Reactivation of Latent Viruses

Studies have associated the reactivation of opportunistic viral infections such as EBV, HHV-6 and CMV with the severity and length of COVID-19 symptoms [43,44]. As previously shown, viruses or their parts may under certain conditions survive the defensive response of the immune system and hide in tissue reservoirs. These latent viruses may then be reactivated, and this reactivation facilitates the entry of the SARS-CoV-2 virus into cells, enhancing viral load and severity of symptoms.

EBV and HHV-6 are the most common immunotropic viruses which use latency after infection to hide from the immune system [44]. EBV has infected more than 90% of the world’s population and more than 95% of healthy adults [45]. Most infected individuals are asymptomatic if they contract it in early childhood. However, patients who contract it during adolescence or adulthood will suffer the symptoms of mononucleosis. Upon the reactivation of EBV, the clinical manifestations include fatigue, brain fog, sleep disturbance, arthralgia, myalgia, pharyngitis, headache, fever, gastrointestinal upset, and skin rashes. Subsequent to the primary bout of infection, the virus enters a state of latency within the B cells to avoid immune surveillance [46,47].

HHV-6 is the collective name for the double-stranded DNA viruses HHV-6A and HHV-6B. More than 90% of humans are infected with HHV-6B within the first three years of life [46]. Those infected with HHV-6 present symptoms that include fatigue, persistent oropharyngitis, recurrent lymphadenopathy, abdominal complaints, and thyroid dysfunction. Sufferers under 3 often develop a skin rash known as roseola. After the primary infection, the HHV-6 virus enters its own latency period, hiding within monocytes, macrophages and the salivary glands [48].

EBV and HHV-6 gain entry into the host cells, particularly B cells, through the oropharyngial epithelium, where they establish latent infection. In the B cell, the viruses undergo intermittent lytic reactivation and replication in epithelial cells, thereby producing infectious virions that are shed in the saliva [49,50]. Because EBV and HHV-6 replicate in the oropharyngial and nasopharyngial epithelium, which is the site of infection for SARS-CoV-2, the subclinical reactivation of EBV and HHV-6 may affect the degree or severity of the SARS-CoV-2 infection [43,51].

The correlation between EBV reactivation and the severity of SARS-CoV-2 infection/symptoms have been confirmed by several studies from all over the world. In order for EBV reactivation to be determined clinically, serological testing for the presence of EBV early antigen-diffuse (EA-D) IgG or EBV viral capsid antigen (VCA) was performed. A study by Simmonet et al. [43] found that in samples from 34 patients admitted to the intensive care unit (ICU) with severe COVID-19 infection, EBV DNA was detected at least once in 28 patients (82.4%). In 19 (67.8%) out of these 28 patients, the amount of DNA was quantifiable with a median viral load of 8648 IU/mL. These patients experienced a longer median ICU stay (15 days) in comparison to those without EBV activation (8 days). The median time for ICU admission of these patients was 4 days following infection, with median time for EBV DNA detection at 7 days following infection. In 22% of the patients, HHV-6 DNA was detected but with viral load being unquantifiable in 6/7 patients. HHV-6 was not linked to a longer ICU stay or a higher simplified acute physiology score (SAPS). Lastly, four (12%) of the total 34 patients presented with a co-occurrence of HHV-6 infection/reactivation [43].

In another study [52], Paolucci et al. used similar methods on a cohort of 104 patients. Overall, 42 of the patients were admitted to the ICU, while the remaining 62 were admitted to the sub-intensive care unit (SICU). All patients but one were positive for EBV IgG (99%). EBV DNA was detected in 40/42 (95.2%) of the ICU patients and 51/61 (83.6%) of the SICU patients. The median level of EBV DNA for ICU patients was 4709 IU/mL, which was significantly higher than the median for SICU patients. Significant reductions in natural killer (NK) cells and CD8^+^ T cells were detected only in patients with the reactivation of EBV but not in those with CMV or parvovirus B19 infections [52].

Just as the previously mentioned studies sought to draw a connection between COVID-19 and viral infections, Gold et al. also attempted to associate EBV reactivation with the extended version of the disease called long COVID in a case study on a cohort of 185 patients [53]. Of the 185, 56 (30.3%) reported long COVID symptoms. Thirty patients were enrolled in a long-term study (90 days and more after COVID-19 diagnosis), and 20/30 (66.7%) tested positive for EBV reactivation as opposed to 2/20 (10%) for the control group. Nine patients were enrolled in a short-term study (21–90 days post-COVID-19 diagnosis), and 7/9 (77.8%) tested positive for EBV reactivation, as opposed to 1/9(11.1%) for the control group. It is noteworthy that only patients positive for EBV EA-D had significant presentations of a number of long COVID symptoms, with the most common being fatigue, insomnia, headaches, myalgia, and confusion [53].

A recent study by Zubchenko et al. came up with very similar results [44]. Among 88 patients diagnosed with COVID-19, 68 (72.3%) tested positive for the reactivation of herpesviruses (EBV, HHV-6, CMV), leaving 20 (27.7%) of the patients without detectable DNA as the control group. Patients with post-COVID symptoms showed a reactivation of EBV in 42.6%, HHV-6 in 25.0%, and reactivation of both EBV and HHV-6 in 32.4%. The 68 patients who were positive for reactivation were more likely to need more intensive care and have a longer median stay in the hospital, 16 days as opposed to 7 days for controls, which was statistically significant. Furthermore, the 68 reactivated patients were more likely to experience elevated ESR, CRP, D-dimer, lymphopenia, monocytosis, and elevated liver enzymes (ALT, AST) [44].

The reactivation of opportunistic viral infections such as EBV, HHV-6 and CMV is common in ICU patients with no previous immune suppression. This reactivation possibly facilitates the entry of the SARS-CoV-2 virus into cells, enhancing the viral load and severity of symptoms, as described by previous studies. While studies regarding the mechanism and true effects of HHV-6 reactivation on long COVID are currently limited, there is clearly a strong correlation between the severity/length of COVID-related symptoms and the reactivation of EBV [14,15,16,17,43,44,53].

Many different reports have discussed the similarity in symptoms and test results of patients with long COVID and patients with autoimmune rheumatic diseases, especially lupus and rheumatoid arthritis (RA). In fact, in patients with RA, anti-EBV antibody response has been found to be strong, leading to the conclusion that the measurement of EBV antibodies might be considered in RA patients as an aid in the diagnosis and the formation of treatment protocols [54,55,56]. Zubchenko et al. concluded that because patients suffering from long COVID who have shown evidence of EBV and HHV-6 have a higher risk of developing various pathologies, including autoimmune disease, such patients should therefore be examined for EBV and HHV-6 reactivation by measuring IgG and IgM antibodies against viral capsid, nuclear, and early antigen [39].

### 3.1. Mechanism by Which EBV Participates in the Enhancement of SARS-CoV-2 Infection

In search of the mechanism by which EBV may enhance or contribute to SARS-CoV-2 infection, Verma et al. [51] found that EBV lytic replication induces ACE2 expression on epithelial cells, facilitating the entry of the SARS-CoV-2 virus into the cells. Additionally, this study found that the ACE2 promoter enhances Z transcriptional activator (Zta) response. Zta promotes the reactivation of the latent EBV virus and the replication of lytic phase EBV. Furthermore, Zta has been found to preferentially act on methylated ACE2 promoters, enabling it to reactivate silenced EBV promoters from their latent state [57,58]. This leads us to believe that EBV infection/reactivation enhances the entry of SARS-CoV-2 into the host cell. The two viruses therefore work hand-in-hand in promoting more severe and prolonged symptoms of COVID-19.

**Figure 4:**
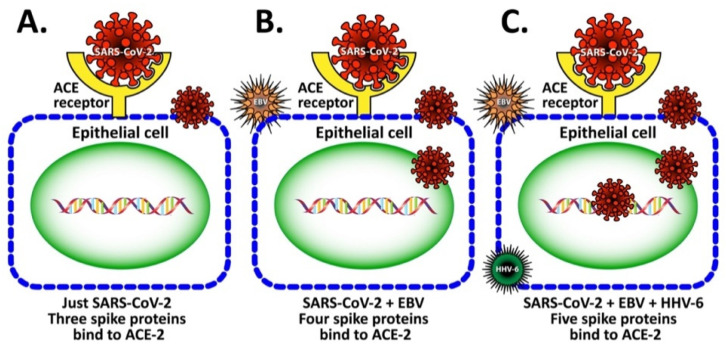
Viral enhancement hypothesis. In this figure, we propose that EBV and HHV-6 lytic replication induces change in ACE expression so as to increase the binding between the SARS-CoV-2 S-protein and the ACE2 receptor, thus enhancing SARS-CoV-2 binding to epithelial cells. (**A**) Just SARS-CoV-2, three spike proteins bind to ACE-2. Infection with SARS-CoV-2 causes a cell expression of ACE-2 receptor that matches with 3 spikes on the SARS-CoV-2 virus. (**B**) SARS-CoV-2 + EBV, four spike proteins bind to AC E-2. Infection with SARS-CoV-2 and EBV causes an expression of the ACE-2 receptor that matches 4 spikes on the virus. (**C**) SARS-CoV-2 + EBV + HHV-6, five spike proteins bind to ACE-2. Infection with SARS-CoV-2, EBV and HHV-6 causes expression of ACE-2 receptor that binds to the virus with 5 spikes for a perfect match.

Whether or not synergism between EBV and HHV-6 further contributes towards ACE2 expression and facilitates SARS-CoV-2 entry into the epithelial cell requires further investigation. We hypothesized that similarly to EBV, the subclinical reactivation of HHV-6 gene expression may enhance the efficiency of SARS-CoV-2 binding to the ACE receptor on human epithelial cells. We propose that in this way EBV, HHV-6 and perhaps other viruses can facilitate the entry of SARS-CoV-2 into the cell. This may be followed by viral replication and the spreading of the virus into the adjacent tissues (see Figure 4). The involvement of HHV-6 in the enhancement of SARS-CoV-2’s entry into epithelial cells should be investigated in future studies. The possibility presented above that two or more viruses (EBV, HHV-6, etc.) may synergistically act as drivers of immunological changes in patients with ME/CFS was previously discussed by Williams et al. [59].

### 3.2. Implication of EBV and HHV-6 dUTPase in ME/CFS and Long COVID

Deoxyuridine triphopsphate nucleotidohydrolases (dUTPases) are metalloenzymes that constitute a new family of pathogen-associated molecular patterns (PAMPs) that are produced by herpesviruses during abortive or productive replication, contributing to pathophysiological changes in herpesvirus-associated diseases [59]. EBV-dUTPase is expressed during the productive replication of the virus as an early BLLF3 gene product and is released in exosomes from the infected and/or stressed cells through a very inflammatory form of cell death called pyroptosis [60,61,62]. After the initial EBV lytic infection, the EBV persists in latent form in an individual’s memory B cell; the reactivation of EBV in the B cell may result in the exosomal release of dUTPase, which may be transmitted to the host’s cells or tissues where it may modulate the cellular microenvironment, thus contributing to the pathophysiological mechanism of EBV-associated disorders [63].

As mentioned earlier, pathogens encode for various PAMPs that are recognized by pattern recognition receptors (PRRs) which act as immune sensors on immune cells. Among PRRs are Toll-like receptors (TLRs), which are responsible for the primary recognition of a variety of pathogens. This leads to the initiation of innate and adaptive immune reactions against many pathogens, including EBV and HHV-6 [64,65,66].

TLR2 is a type of receptor that is localized on the surface of immune cells and can form complexes with TLR1 or TLR6. A significant binding affinity exists between viral dUTPases (PAMPs) and TLR2. Although TLR2 is part of the innate immune response and is vital for the defense against viruses, its activation has been shown to be involved in various autoimmune diseases, including RA, Sjögren’s syndrome, MS, systemic sclerosis and systemic lupus erythematosus (SLE) [67]. This is because once engaged, TLR2 activates a signaling cascade that results in the production of proinflammatory cytokines that contribute to the development and progression of many disorders [68,69,70]. For example, Ariza et al. demonstrated that through the engagement of the TLR2 homodimer, EBV-dUTPase induces the activation of NF-κB, which is a very strong proinflammatory mediator [71,72]. Interestingly, HSV-2 and HHV-6A dUTPases also activated NF-κB after ligation with TKR2/1 heterodimer [68,69]. Furthermore Ariza et al. showed that EBV, HHV-6 and other viral dUTPases are capable of activating different lymphocyte subpopulations and inducing the production of T-helper-1 (Th1), T-helper-17 (Th17), and other inflammatory cytokines such as IL-1β, IL-6, IL-8 and TNF-α [71,72,73].

It is interesting to note that EBV and HHV-6 dUTPases have differences in their abilities to induce cytokine secretion and NF-κB activation, which may be a reflection of the differences of their molecular sizes, amino acid sequences, and their binding affinities to TLR2 or other TLRs [68,69,70]. For example, the degree of identity between EBV and HHV-6 A and B dUTPases was shown to be 21%, but with HHV-8 dUTPase, it was 31% [59]. This protein homology between EBV and HHV-6 dUTPases may be the reason that about 20% of ME/CSF patients co-expressed antibodies against EBV and HHV-6 dUTPases, and 28.41% produced antibodies against EBV, HHV-6, and human dUTPases [74]. These results indicate that the reactivation of multiple herpesviruses (EBV, HHV-6) and their production of dUTPase can elicit both humoral and cellular immune responses, including the hyperactivation of Th1 and Th17 in some patients with ME/CSF [71,72,73]. It is entirely possible that this also applies to patients with long COVID, because many of the symptoms and conditions observed in long COVID are also shared by patients with ME/CSF [75,76].

**Figure 5:**
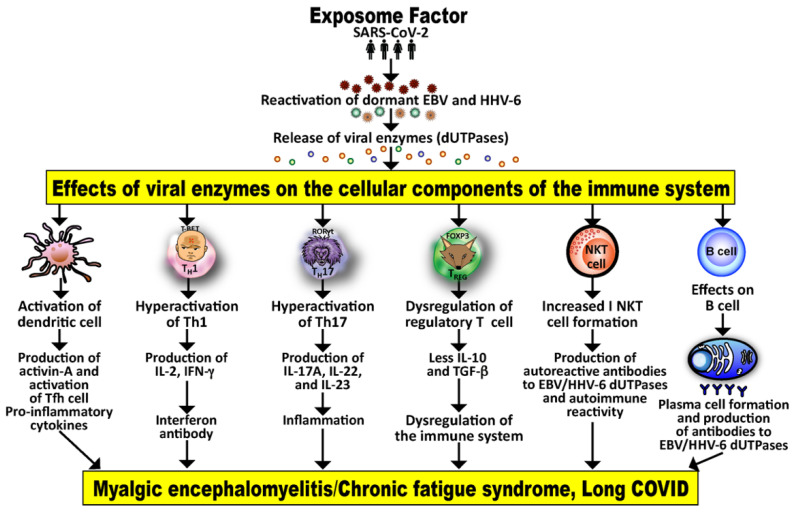
The exposome factor or lifetime exposure to internal and external triggers (in this example, specifically SARS-CoV-2) may affect the reactivation of dormant viruses and cause the release of their viral dUTPases, which in turn can affect the humoral and cell-mediated components of the immune system, resulting in the activation of dendritic cells, Th1, Th17 and NKT cells. This may result in the production of many inflammatory cytokines and antibodies that together contribute towards ME/CFS and long COVID.

The exposome is a person’s lifetime exposure to internal and external factors [6,77]. It may cause the reactivation of multiple herpesviruses (EBV, HHV-6), causing the release of their different dUTPases. These may in turn affect the different components of both the humoral and cell-mediated immune system, possibly causing deficiencies, abnormalities or dysfunctions that may result in long COVID or ME/CFS in some patients. The mechanism for this is shown in Figure 5.

Because the release of dUTPase by both EBV and HHV-6 results in immune reaction and the production of antibodies, measurements of IgG and IgM antibodies against these viral antigens should be investigated.

## 4. Viral Superantigen Activation of the Immune System

Superantigens (SAgs) are a class of antigens that are produced by some pathogenic viruses and bacteria as a defense mechanism against the immune system. Because they cause the non-specific activation of T-cells, they induce polyclonal T cell activation and massive cytokine release, resulting in excessive activation of the immune system, which may lead to autoimmunity, multiple organ failure, and even death [78]. They are known to elicit a very strong immune response and the release of proinflammatory cytokines by activated T cells through the cross-linking of receptors on those T cells with MHC class II molecules [79,80]. Some SAgs can also hyper-stimulate B cells into producing heightened levels of antibodies [78].

In relation to SAgs and SARS-CoV-2, in a very recent perspective by Hamdy and Leonardi [78], it was shown that SARS-CoV-2 contains a very unique superantigen-like peptide not found in any other SARS virus. This SARS-CoV-2 (671–692) SAg-like motif is shown below:

CASYQTQT_NSPRRARSVASQSI

However, in their conclusion, it is stated that “It is of vital importance to definitively establish whether SARS-CoV-2 is a superantigen, superantigen-like, or triggers a superantigenic host response in order to better understand the short and long-term consequences of infection” [78]. The reason for this classification is the identification of the SAg motif close to the SARS-CoV-2 S1-S2 cleavage site of spike protein with a similar sequence to *Staphylococcus* enterotoxin B (SEB), which is known as a classical SAg [79].

To further support this homology between the SARS-CoV-2 SAg motif of S protein and SEB, monoclonal antibodies were screened for their ability to bind to this motif. The SEB monoclonal antibody not only bound to the SAg motif on the S protein but was capable of inhibiting infection with live SARS-CoV-2 [79]. Furthermore, Cheng et al. [79] showed that this superantigenic character of a unique insert to SARS-CoV-2 exhibited a high affinity for binding to the T cell receptor (TCR) that may form a complex with MHC class II. The binding of this SAg to the TCR may trigger the cytotoxic adaptive immune responses observed in multi-system inflammatory syndrome in children (MIS-C) as well as cytokine storm in adults with SARS-CoV-2 infection [79].

Molecular chaperones such as heat shock proteins (HSPs) are another group of proteins which share epitopes with SARS-COV-2 that potentially are capable of eliciting autoimmunity [81]. HSP proteins, which make up approximately 5–10% the total proteins in most cells, are categorized based on their molecular weights. HSP10 and HSP21 are classified as small, HSP60, HSP65 and HSP70 are known as medium, and HSP90 and HSP100 are classified as large [82]. Overall, the main role of HSPs is the protection of cellular components against stressors by maintaining the correct folding of newly synthesized proteins, participating in the repair of misfolded proteins, and transporting proteins to their site of action [83]. In the absence of stressors, HSPs are associated with a specific factor called heat shock factor (HSF). However, cellular stress and an increase in protein damage results in the release of HSP from the HSF complex in order to bind to damaged proteins. The free HSF in cells manage to bind to heat shock DNA elements, resulting in HSP expression, which is called heat shock response or HSR [82,83].

Viruses in general lack classical HSPs, so they use their hosts’ HSPs to fold their proteins, which contributes to the effectiveness of viral infection. The HSPs also support viruses by forming complexes on the cell surface to enhance the viruses’ entry into the host cells. Furthermore, they interact with viral polymerases to support viral replication [82]. Finally, it has been shown that HSPs share a significant homology with SARS-CoV-2 antigenic epitopes, which could generate immunological cross-reactivity in COVID-19 that might be the cause of autoimmunity [81]. HSP60, HSP65, HSP70 and HSP90 are four major proteins that share homology not only with other human proteins but also with many viruses, including SARS-CoV-2 [64,78,79]. The immunological relevance of this peptide sharing between human HSPs and SARS-CoV-2 proteins is shown in Table 1.

Through different mechanisms, both the viral and the host’s superantigens seem to contribute to persistent SARS-CoV-2 infection that may result in long COVID. By the over-stimulation of immune responses, the HSP-like molecules and other superantigens of the virus induce a negative feedback loop that allows SARS-CoV-2 to persist in the tissue. Normally, the presence of host factors would induce the activation of the innate and adaptive components of the immune system, resulting in a biased T-helper cell response that would influence fast recovery from viral infection [84]. However, THR cell imbalance following infection with the virus, particularly the over-activation of Th1 and Th17, could contribute toward long COVID. This provides justification for using a patient’s lymphocyte map results to design treatment modalities. This emphasis on the Th cell status of patients with viral infection may prove to be the most effective strategy for the treatment of patients with long COVID. Furthermore, the innate and adaptive immune responses against viral superantigens result in high levels of antibodies against them, which is why further investigation of the possible roles for these viral superantigens in long COVID is warranted.

## 5. Disturbance in the Gut Microbiota

A microbiome is a community or collection of microorganisms that co-inhabit a particular environment such as the human body or a part thereof. These microbes include bacteria, bacteriophages, protozoa, fungi, viruses and helminths [85]. Largely unnoticed and outwardly invisible, these living organisms co-exist in a stable relationship within a healthy human body and clearly exert considerable influence on all aspects of our physiology. However, as mankind has evolved, so, too, has the microbiome. The microbiome of twenty-first century man is vastly different from that of his forebears, because the food, living conditions and daily experiences of modern man have been changed by literal millennia from that of primitive man [86].

The transition from living in the wild to communal urban living, continuing technical progress, industrialization, and modernization throughout the ages have brought about lifestyle changes such as increases in hygiene and sanitation, antibiotics and the consumption of processed foods. All this has unavoidably affected the composition of our bodies’ microbiomes [87].

We know that environmental factors can induce autoimmune responses and can interact with the diverse teeming occupants of our microbiome. We can readily understand, then, that in genetically predisposed or susceptible individuals, dysbiosis or disturbances in the gut, oral and skin microbiomes have been linked to damage to the tissues and actual autoimmunity. This is because alterations in the microbial composition of a microbiome induce inflammation and a failure in immune tolerance, and they could ultimately contribute significantly toward autoimmunity. Gut microbial changes such as this are the hallmark of COVID-19, particularly long COVID [48,87,88,89,90].

In relation to microbiome changes in COVID-19, Dotan et al. in 2022 published a review article [91] in which they described “COVID-19 as an infectome paradigm of autoimmunity.” They concluded that the reviewed evidence showed that the microbiome, particularly the gut and lung microbiome, may have a pivotal role in the pathogenesis, clinical severity, outcome, and possibly even treatment of COVID-19.

Wang et al. focused on the oral, gut, and lung microbiomes when they reviewed the microbial characteristics of COVID-19 [92]. They found that in comparison to that of healthy subjects, the microbial composition of COVID-19 patients was significantly changed, especially the microbiota of the gut and lungs. This suggests that these microbiota may be useful as biomarkers for COVID-19 and acute respiratory distress syndrome (ARDS).

Yeoh et al. made some interesting findings regarding gut dysbiosis when they compared the gut microbiomes of COVID-19 patients with those of healthy controls [93]. They found that the feces of COVID-19 patients had a lower number of *Roseburia, Faecalibacterium prausnitzii, Eubacterium* and *Lanchnospiraceae*, which are known as anti-inflammatory bacteria, but they were enriched with *Enterococcus, Enterobacteriaceae, Clostridium hathewayi, Bacterius nordii* and *Actinomyces viscosus*, which are known to cause bacteremia. This sort of fungal gut microbiome has been observed in response to infection with SARS-CoV-2 [94,95]. This indicates that the dysbiosis of gut fungi may contribute to fungal infection in COVID-19 patients. There is substantial evidence that the perturbance of these microbiota is associated with the induction and disease severity of long COVID or post-acute COVID-19 syndrome (PACS) [91,96,97,98,99].

In a recent study [96], Liu et al. provided observational evidence of gut dysbiosis in patients with long-term sequelae after COVID-19 infection. For their study, the researchers analyzed a serial fecal microbiome of 258 samples using shotgun metagenomic sequencing. They then correlated the patients’ gut microbiota changes with persistent symptoms at 6 months. Based on the results, they first showed that the gut microbiota composition at the time of hospital admission was associated with the occurrence of long COVID. Secondly, they found that the gut microbiome profile at 6 months of patients with SARS-CoV-2 infection but without long COVID was comparable to those of controls who had not contracted COVID-19. Thirdly, they demonstrated that the gut microbiome of patients with long COVID was characterized by higher numbers of *Bacteroides vulgatus, Ruminococcus gnavus, Erysipelatoclostridium ramosum, Actinomyces johnsonii, Atopobium parvulum* and *Clostridium innocuum* that positively correlated with long COVID.

**Figure 6:**
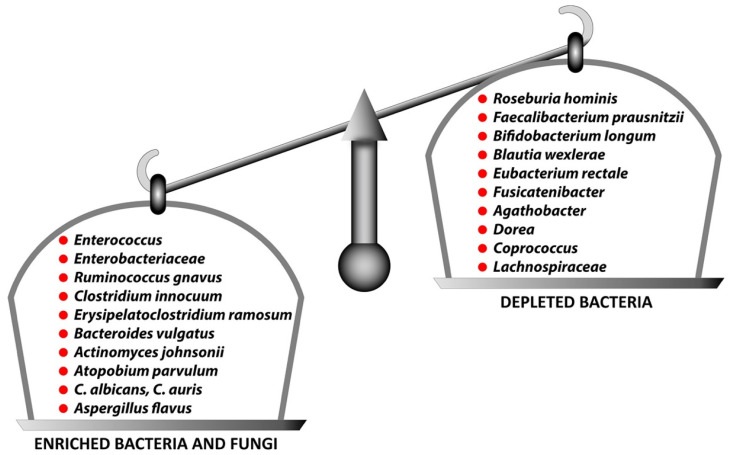
Enriched bacteria and fungi positively associated with long COVID or PACS versus depleted bacteria negatively associated with long COVID.

Moreover, patients without long COVID had gut bacterial compositions that were enriched for 19 bacteria, characterized by *Bifidobacterium, Brautia* and *Bacteroidetes.* At 6 months, 13 bacterial species including *Blautia wexlerae* and *Bifidobacterium longum* were negatively associated with long COVID. This indicates that these species may have protective roles during recovery from COVID-19. A summary of this dichotomy of these two positively or negatively associated groups noted by Liu et al. [96] and reviewed by Wang et al. [92] is shown in Figure 6.

Because disturbance in the gut microbiota has been associated with the activation of monocytes and neutrophils, the production of S100A12, damage to the tight junction and the induction of leaky gut, measurements of microbiome RNA, DNA, direct stool culture, zonulin, zonulin antibody, IgG/IgM/IgA antibodies against various bacterial toxins should be investigated in long COVID patients.

These discoveries strongly suggest that the modulation of gut microbiota by the use of medication, microbial transplantation, probiotics, prebiotics, engineered symbiotic bacteria, gut commensal-derived metabolites, amino acid derivatives, carbohydrates, vitamins, polysaccharides, glycolipids and more could facilitate faster recovery from COVID-19 and reduce the risk of long COVID development [92,96].

## 6. Multiple Tissue Damage and Autoimmunity

Autoimmune diseases can be recognized and classified by certain characteristics, such as the presence of detectable autoantibodies. The disruption of the immune system and breakdown in immune tolerance can result not only in long-term inflammatory reactions but malfunction and actual damage to organs that fall victim to mistaken immune responses [100].

The potential role of autoimmunity in the induction of long COVID was discussed in the earlier sections in the context of viral persistence, reactivation of herpesviruses (especially EBV and HHV-6), viral superantigens, and gut microbiota. We see that different mechanisms including molecular mimicry between components of viral antigens and various human tissue antigens can lead to the immune system becoming chronically activated, thus inducing autoimmune responses [101,102,103,104]. One such antigen that shares structural similarity with SARS-CoV-2 and herpesviruses is neuronal protein [83,84,86]. It has been found that both COVID-19 and long COVID share the presence of certain autoantibodies with a form of autonomic dysregulation called postural orthostatic tachycardia syndrome (POTS) [100,105]. These autoantibodies are known to attack G-protein-coupled receptors on neurons [106]. Many pathogens, including viruses and bacteria, contain superantigens that are capable of activating T and B cells in a non-specific manner, producing a variety of autoantibodies in patients with multisystem inflammatory syndrome and patients with acute COVID-19 [107,108]. Finally, the reactivation of herpesviruses, particularly EBV and HHV-6 [29,30] that not only share a significant homology with SARS-CoV-2 spike protein [4,103] but co-infection with these viruses may change the structure of the ACE2 receptor on the epithelial cell in such a way that SARS-CoV-2 will complete its infective cycle much more efficiently (see Figure 4). In fact, in a subgroup of patients with long COVID, very high levels of antibodies against EBV and HHV-6 were reported [44]. This may be why some clinical features of moderate to severe SARS-CoV-2 infection are mimics of those seen in lupus, inflammatory arthritis, anti-phospholipid syndrome, and other autoimmune diseases [109,110,111,112,113,114]. Moreover, there are many reports of patients who, following infection with SARS-CoV-2, developed classical autoimmune diseases such as cardiomyopathy, type 1 diabetes, rheumatoid arthritis, psoriatic arthritis, lupus, idiopathic inflammatory myopathies, systemic Guillain–Barré syndrome, thyroid autoimmunity, sclerosis and more [115,116,117,118,119,120,121,122,123,124,125].

These autoimmune conditions that followed SARS-CoV-2 infection were identified based largely on specific autoantibodies that were detected in the blood of the patients. Although more research is still emerging, many researchers believe that severe SARS-CoV-2 infection can lead to new potentially pathogenic antibodies that may attack host tissues and cause harm [114,115,116,117,118,122,123,124,125]. These autoantibodies may enhance different symptomatologies that are increasingly observed in long COVID patients [103]. The possible contributions to long COVID may stem not only from mimicry between SARS-CoV-2 and a variety of tissue antigens [91,103] but also from the reactivation of latent viruses such as EBV and HHV-6 in COVID-19 patients and their synergistic effects in the development of autoimmunities.

These three viruses are rapidly becoming strongly associated with autoimmunity. However, due to its many connections and contributions to autoimmune diseases, and, of course, its glaring presence in the public eye, extra focus has particularly been brought to bear on the SARS-CoV-2 virus. Ever since the outbreak of COVID-19, more and more evidence continues to be gathered about SARS-CoV-2’s associations with autoimmunity and immune dysregulation [102,103,126,127,128,129,130,131,132,133,134,135,136]. In a subgroup of patients with SARS-CoV-2 infection, the detection of certain autoantibodies and circulating inflammatory mediators correlate with the report of symptoms related to autoimmunity and inflammation in individuals presumed to be genetically pre-disposed [102,103,126,127,137,138,139,140].

The following previously published key points [102,103,131] that were also presented by the corresponding author at the 13th International Congress of Autoimmunity [141] support the concept of SARS-CoV-2 being one of the autoimmune viruses:

1. SARS-CoV-2 spike proteins and nucleoproteins share molecular mimicry with human autoantigens involved with autoimmune diseases;
2. Both animal and human monoclonal antibodies (mAbs) made against SARS-CoV-2 spike proteins and nucleoproteins react with human autoantigens;
3. Antibodies made against human autoantigens react with SARS-CoV-2 spike proteins and nucleoproteins;
4. The sera of patients with COVID-19 have tested positive for autoantibodies made against human autoantigens known to cross-react with SARS-CoV-2.

These four key points are the best experimental evidence that was generated in our lab for SARS-CoV-2 being one of the “autoimmune viruses” [102,103,131].

### 6.1. Herpesviruses and the Pathophysiology of Autoimmunity and Long COVID

**Figure 7:**
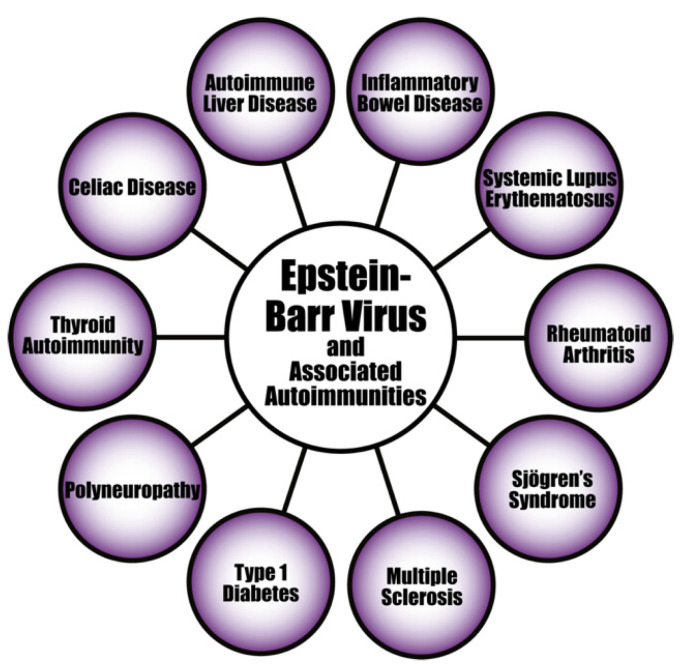
Epstein–Barr virus and a few of its associated autoimmune diseases.

Evidence continues to mount linking the development of different autoimmune disorders to herpesvirus infections (see Figure 7). It has been shown, for example, that the pathogenesis of systemic autoimmune diseases (SADs) can be triggered by the reactivation of latent herpesviruses [142,143,144,145]. In the case of SADs, attention is especially being focused on EBV infection as playing an important role in their pathogenesis.

#### 6.1.1. EBV

Researchers have found increased EBV viral mRNA expression and elevated viral loads of EBV DNA in the blood of SLE patients [146,147,148]. Abnormal cell-mediated immunity, increased EBV viral load, and high levels of EBV antibodies have also been demonstrated in patients with RA and Sjögren’s syndrome [145,149].

EBV can also lead to autoimmunity through molecular mimicry when autoantigens associated with lupus in SLE patients cross-react with antibodies produced against EBNA-1. The resultant epitope spreading could cause this cross-reactivity to expand to even more autoantigens [150]. TLR3 signaling is another way in which EBV infection can induce the activation of innate immunity, leading to the production of proinflammatory cytokines and interferon [151]. Further evidence for EBV reactivation as an environmental trigger of autoimmunity is provided by the serological detection of IgG early antigen antibodies in individuals with established lupus that correlate not only with transition to SLE but also with severity of the disease [152,153]. Additionally, antibodies made against EBV nuclear antigen cross-react with human Sm and Ro that are known to be lupus-associated antigens, and they correlate with these lupus-associated autoantibodies [153,154,155,156,157,158,159]. Interestingly, due to viral reactivation, the same EBV antibodies that were detected in SADs were shown to be elevated in long COVID patients [26,43,44,51,53]. Thus, EBV and other herpesviruses, such as HHV-6, could be the common denominators between long COVID and autoimmunity.

#### 6.1.2. HHV-6A and HHV-6B

**Figure 8:**
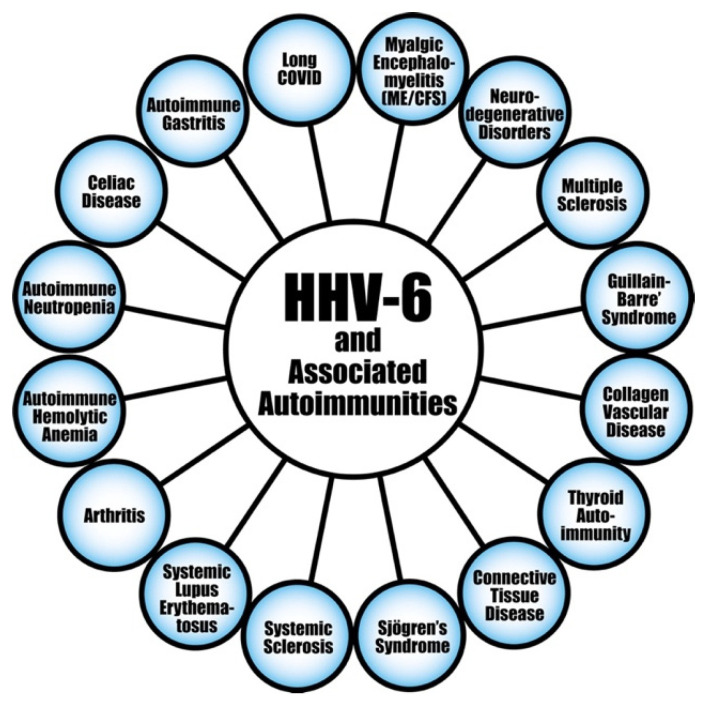
HHV-6 virus and a few of its associated autoimmune diseases.

HHV-6 is another member of the herpesvirus family that is believed to play a major role in the induction of autoimmunity. Like EBV, it also has the capacity to sometimes escape elimination, lie low, and wake like a sleeper cell for reactivation. By carrying stretches of telomeric repeats at their linear genome, the variants HHV-6A and HHV-6B are able to integrate into human chromosomes and become latent [160]. This virus has been found in several human diseases, including autoimmunity [161,162,163,164,165,166,167,168,169,170,171,172,173,174,175,176,177,178,179,180,181,182,183,184,185,186,187,188,189], ME/CFS [190,191,192,193], mitochondrial dysfunction [193,194], and in patients with moderate to critical COVID-19 [42,43,44,195,196]. Among the autoimmune diseases associated with HHV-6, the most frequently cited is multiple sclerosis (MS) [170,171,172,173,174,175,176,177,178,179,180,181,182,183,184]. This has been demonstrated by the direct detection of the HHV-6 genome and antigens in the blood and brain of patients with MS [176,177,178,181,182,183]. Furthermore, not only were higher levels of anti-HHV-6 A/B antibodies detected in the blood of MS patients, their titers also correlated with the severity of the disease [170] and in predicting risk of relapse [184,185,186]. Researchers further concluded that in addition to its potential etiologic role in MS, infection with HHV-6 or immune reaction against it may also have an effect on MS relapse and its progression [184]. Because this observed clinical effect is directly related to anti-HHV-6 antibody titer, the measurement of antibodies to HHV-6 antigens may be a useful prognostic factor in the clinical course of MS [184]. In addition, several reports have provided important data connecting HHV-6 A/B with other autoimmune diseases, including RA [156], collagen vascular disease [162], lupus [163], connective tissue disease [164,165], Sjögren’s syndrome [166], systemic sclerosis [167], thyroid autoimmunity [168,169], severe and acute autoimmune hepatitis [187], autoimmune hemolytic anemia [188], and autoimmune neutropenia [189]. Moreover, in a recent study, Bigley et al. [197] observed that adult mice infected with murine roseolovirus (MRV), which acts like HHV-6 and HHV-7 during the neonatal phase, developed autoimmune gastritis (AIG). It is notable that this autoimmune phenomenon developed months after resolution of acute viral infection. In search of the mechanism, the researchers found that there had been a breakdown in the central tolerance that was dependent on CD4+ T cells and IL-17-producing cells, resulting in an attack on the stomach tissue, and, thus, the development of autoimmune disease [197]. This induction of autoimmunity by MRV was accompanied by the production of autoantibodies against antigens, some of which are typically associated with AIG, and some which are not: anti-H+/K+ ATPase (typically detected in AIG) and other autoantibodies such as thyroglobulin, thyroid peroxidase, insulin, double-stranded DNA, topoisomerase 1, glomerular basement membrane, and myoloperoxidase, which are detected in many autoimmune diseases. Based on this and other data, Bigley et al. concluded that the murine equivalent of human HHV-6 and HHV-7 induced a significant breakdown in the central tolerance mechanism that resulted in the production of antibodies against a widespread variety of autoantigens that are detected mainly in polyautoimmunity [197]. These findings about the presence of so many autoantibodies in the blood of mice infected with MRV may explain the involvement of HHV-6 in many autoimmune disorders (shown in Figure 8) through a completely new mechanism which involves the disruption of central tolerance [197].

Although it is already very well-known how viruses, including HHV-6, through molecular mimicry, bystander activation, epitope spreading, and activation of autoreactive Th1 and Th17 cells induce various autoimmune diseases [162,163,164,165,166,167,168,169,170,171,172,173,174,175,176,177,178,179,180,181,182,183,184,185,186,187,188,189], the breakdown of thymic central tolerance by viruses such as HHV-6 and HHV-7 is a novel one.

Altogether, we may conclude that the reactivation of latent EBV, HHV-6 and possibly other viruses may act synergistically with SARS-CoV-2 to pre-dispose a patient toward autoimmunity, which is a hallmark of long COVID or post-COVID syndrome. In support of this hypothesis, very recently, Rojas et al. [198] demonstrated that latent autoimmunity was detected in 83% and polyautoimmunity in 62% of patients with post-COVID syndrome.

The awareness of the association connecting herpesviruses, SARS-CoV-2 and long COVID creates new opportunities for the diagnosis, management, and possible treatment of this new disease [53].

Thus, in addition to measuring IgG and IgM antibodies against reactivated latent viruses such as EBV, HHV-6, CMV, heat shock proteins and other viral superantigens, it is highly recommended the measurement of antibodies against type 1 interferon, S100A12, ANA, ENA, dsDNA, RF, actin, mitochondrial antibodies, C1Q immune complexes and other antigens in the blood of patients with long COVID should also be investigated.

Furthermore, aside from the aforementioned autoantibodies, the possible contribution to long COVID of autoreactive T cells warrants their scrutiny, especially the relationship and balance/imbalance between Th1/Th17 and regulatory T cells. Thus, the determination of the composition of the lymphocyte subpopulation by flow cytometry may shed light on the pathophysiological changes in a subset of patients with long COVID 9 [199].

## 7. Treatment: Targeting Latent Viral Reactivation and Microbiota Manipulation as Strategies for the Prevention and Treatment of COVID-19 and Long COVID

Currently, there are several medications for targeting SARS-CoV-2 directly. These include Paxlovid (Pfizer, New York, NY, USA), Lagevrio (Merck, Rahway, NJ, USA), Remdesivir, Ivermectin, Chloroquine, Hydroxychloroquine, and Bebtelovimab.

Zinc plus low-dose hydroxychloroquine and azithromycin was suggested by Derwand et al. [200] for COVID-19 outpatients.

Since 2020, Ivermectin had been suggested or even used in many countries as a potential chemoprophylaxis against COVID-19 [201]. It has also been shown very recently as an effective treatment against SARS-CoV-2 through its feeding of *Bifidobacterium* and increasing its number. On the one hand, this increase in bacterial levels boosts natural immunity; on the other hand, it reduces the production of inflammatory cytokines, but this is actually also a benefit, since these cytokines contribute toward a cytokine storm, so that Ivermectin has two ways to protect against COVID-19 [202].

Moreover, the use of nicotinamide adenine dinucleotide (NAD^+^) in COVID-19 and viral infections in general has been shown to suppress NF-κB and NLRP3 inflammasome activity. This nicotinamide seems to contribute to the resolution of inflammation, limiting the effects of cytokine storms observed in COVID-19 and possibly other viral infectious diseases [203].

In relation to targeting microbiota disturbance for the prevention and treatment of COVID-19, there are many immunomodulatory options that are briefly discussed in this manuscript [96]. This includes dietary intervention, prebiotics, probiotics, short-chain fatty acids (acetate, butyrate, propionate) that are produced by gut microbiota after feeding on fibers, AHR blockers, vitamins A and D (these bind to receptors on CD4+CD25+ cells with a capacity to regulate the immune system in the gut and beyond), and potential nutritional and antioxidant supplements. The use of polyphenols, probiotics, vitamin D and omega-3 fatty acids was suggested by Daoust et al. [204]. Fecal transplant, microbiota transplantation, probiotics, prebiotics, microbiome-derived metabolites, and engineered symbiotic bacteria as treatment options that could modulate host microbiota and mitigate virus-induced inflammation in the gut have been suggested by different studies [205,206,207,208].

KB109 is a synthetic glycan that helps to support the homeostasis of the immune system through microbiome modulation in patients with mild to moderate COVID-19. In COVID patients with comorbidities, this product reduced medically attended acute care visits and shortened symptoms [209].

In addition to SARS-CoV-2′s alteration of gut microbiota, the enrichment of opportunistic pathogens and depletion of beneficial commensals which produce SCFA, SARS-CoV-2 also invades enterocytes via ACE2, which may cause disturbance of the gut microbiota and leaky gut syndrome [102]. Although ACE2 is known as a maintainer of the balance of the renin–angiotensin system, it is also a key regulator of innate immunity, microbial balance, and dietary amino acid homeostasis [210]. During a SARS-CoV-2 infection, the virus invades the gut by binding to ACE2, and it undergoes replication to promote and spread the viral infection [211,212]. This reduction in ACE2 by the virus leads to the accumulation of angiotensin II, the ligand for ACE2, resulting in the overproduction of endotoxins which contributes to enhanced intestinal permeability to macromolecules, and, thus, to leaky gut [213]. This impairment of barrier function and the entry of lipopolysaccharides into the systemic circulation may contribute to hyperactivation of the immune system and an overproduction of inflammatory cytokines that activate the inflammatory cascade.

Beneficial effects against COVID-19 and long COVID can even come from very ordinary, commonplace things. In a 2022 review, Foolchand et al. state that vitamin D and its receptor not only fight viral infection in general, but that they also function to regulate genes involved in immunity, apoptosis, proliferation, differentiation, and inflammation. These immunomodulatory effects of vitamin D suggest a role for it in the treatment of COVID-19 [214]. Even a seemingly ordinary practice such as dieting or fasting may have a great beneficial effect for immunity against COVID-19. Fasting causes the synthesis in the liver of the ketone body β-hydroxybutyrate (BHB). BHB is a multifunctional molecule that not only provides energy fuel but also exerts direct effects on immune cells [215]. Karagiannis et al. compared peripheral blood from patients suffering from acute respiratory distress syndrome (ARDS) induced by SARS-CoV-2, influenza or bacterial respiratory infections, and found that patients with COVID-19 had substantially lower serum concentrations of BHB, indicating dysregulated ketogenesis [215]. They also observed that BHB supplementation increased cell numbers and enhanced IFN-γ production in human and mouse Th1 cells under amino acid-deficient conditions in vitro. The oral administration of ketone esters improved antiviral CD4^+^ T cell functional fitness and viral clearance, resulting in benefits that included improved overall survival. This research suggests that BHB is a potential therapeutic target for the treatment of severe COVID-19 and provides new insights and understanding of dietary influence on antiviral immunity against COVID-19 [215].

There have even been attempts to find natural means and treatments to combat COVID-19. Pérez-Vargas et al. sought to explore a novel spectrum of antivirals that would be effective against both existing and the seemingly endlessly evolving SARS-CoV-2 variants of concern (VOCs). They screened a library of natural products (NPs) obtained from plants, fungi, bacteria and marine sponges and achieved promising results, finding that NPs with highly diverse chemical structures have the potential for being developed into SARS-CoV-2 therapeutics [216].

However, it is well known that even in the presence of treatment for SARS-CoV-2, a significant proportion of patients develop many symptoms that persist up to more than 8 months after the initial infection [217]. This is because in addition to SARS-CoV-2, the reactivation of latent viruses such as EBV, HHV-6, CMV and possibly unknown factors are responsible for the development of long COVID in a subgroup of patients [27,43,44,51,53,217]. In fact, Gold et al. concluded that “long COVID symptoms may not be a direct result of the SARS-CoV-2 virus, but may be the result of COVID-19-induced EBV reactivation” [53]. Further evidence that EBV and other herpesviruses may be involved in long COVID comes from treatment with ganciclovir; this known anti-herpesvirus medication has the capacity to block EBV replication, and treatment with it reduced the risk of death of severe COVID-19 patients [218]. In addition, there have been attempts to reduce the viral load in the reactivation of herpesviruses by using anti-DNA viral agents. These drugs have shown some measure of efficacy in the management of EBV disease. For instance, the extended administration of valacyclovir has been shown to reduce the frequency of EBV-infected B cells and is being proposed as a protocol for eradicating EBV from a host [219]. EBV VCA synthesis and capsid formation have been found to be inhibited by Spironoloactone in vitro [220]. Its effectiveness has led Spironolactone to being considered as a potential treatment for SARS-CoV-2 infection itself [221,222].

The medications and treatment protocols for COVID-19 have been rushed through formation and implementation in response to the initial desperate and immediate need, so that they are now fairly well-known and more or less available. In comparison, actual treatments for long COVID are less researched, less known, and less obvious, but they do exist. It is logical to posit that many of the treatments and medications currently being used for the management of COVID-19 have the potential to be used against what is essentially the long-drawn out extended version of the disease known as long COVID. Molnupiravir (Lagevrio) was one of the first oral medications to be approved for treating mild to moderate COVID-19. A recent preclinical study found that molnupiravir attenuated chronic long COVID symptoms [223]. The National Institutes of Health (NIH) is also testing several known immunomodulatory drugs, including the monoclonal antibody Infliximab, which is approved for the treatment of Crohn’s disease, for possible use against long COVID [224,225]. Studies show that antihistamines appear to provide relief for long COVID sufferers [226,227].

Treatment is intended to maximize a patient’s functions and improve the quality of life [228]. In the absence of clinical trials, treatment recommendations for long COVID are based on consensus guidelines and the opinions of experts [228,229,230,231,232,233,234,235,236]. A comprehensive treatment protocol must consider a total patient viewpoint that encompasses the patient’s symptomatology, any pre-existing conditions, psychological, social and personal well-being, and the treatment’s specific goals [10,228].

Many of long COVID’s associated symptoms and conditions are already commonly seen in primary care practice and can be treated with established therapeutic protocols and the proper supportive care [228,232,234].

If long COVID has affected the patient to the point of cognitive impairment, a specialist in the appropriate neuro-associated field should be consulted [235].

Monoclonal antibodies against SARS-CoV-2 have been suggested to be used in treatments against both COVID-19 and long COVID. In a small pilot study, researchers found that the CCR5-binding monoclonal antibody leronlimab was able to improve symptoms in some individuals suffering from long COVID apparently by boosting their immunity [237].

The research group of Gil et al. found that patients suffering from long COVID (under the name PASC) with one or more persistent symptoms were associated with physical inactivity. They proposed that their findings may help PASC patients by tailoring treatments to include combating inactivity, with potential overall health benefits [238]. For their part, Scurati et al. found in their own review that exercise is known to exert a deep action on molecular dysfunctions elicited by long COVID-19. Of course, it depends on training, intensity, duration and continuity, but the information they presented bears the consideration of properly regulated exercise as part of the management protocol for long COVID [239]. For patients exhibiting the fatigue or post-exertional malaise characteristic of both long COVID and ME/CSF, a personalized, symptom-guided program for a phased return to activity is recommended [230].

Considering that we have just described a vital role in long COVID for the reactivation of herpesviruses and disturbance of the gut microbiota, targeting SARS-CoV-2 herpesviruses and gut microbiota has been suggested for the prevention and treatment of long COVID. Although many patients may experience the resolution of some or even all of the various symptoms and conditions of long COVID over time, it should be noted that the actual effective treatment and prognosis of the disease is still far from definitive at this point [234]. It is obvious that using therapy that combines all that we have detailed above, anti-viral and pharmacological agents, bacterial transplantation, prebiotics, probiotics, mAbs, and dietary intervention, all these measures that strengthen the immune system can only be of benefit for individuals suffering from long COVID, alleviating the severity of the disease and improving their recovery time [102,240,241,242,243,244,245,246,247,248,249,250].

## 8. Conclusions

It is now known that in some individuals, COVID-19 can lead to a long-term disease that has been called many names but is most commonly referred to as long COVID. The many different symptoms and conditions associated with long COVID and their lasting implications and consequences have still to be fully understood, with patients ranging from being asymptomatic to requiring hospitalization and even intensive care [251,252,253,254].

Data show that from 10 to 30% of the hundreds of millions of people who had acute COVID-19 progressed to long COVID, with the CDC reporting that 19% of adults who had COVID-19 are still suffering from symptoms [7,229,237,255].

What causes some patients who suffer from COVID-19 to proceed to long COVID? There are three stages or phases that have been characterized that not all COVID-19 sufferers go through [256]:

- Phase 1. Acute COVID-19 with varying degrees of severity caused by viral replication and initial immune response. This can last from days to weeks, and asymptomatic patients have also been known to progress to the later phases [256].
- Phase 2. Two to five weeks after the onset of the infection, a rare hyperinflammatory condition known as multisystem inflammatory syndrome may occur, with signs and symptoms similar to Kawasaki disease. This is caused by a dysregulated immune response and can affect both children and adults [256,257,258,259].
- Phase 3. Long COVID may develop and persist for months with varying symptoms [7,10,228,260].

However, what would make it more likely for a COVID-19 patient to go through all three of these phases into long COVID? The following are some of the risk factors for long COVID as shown by studies: type 2 diabetes mellitus, SARS-CoV-2 viremia, EBV viremia, certain specific autoantibodies, 50+ years in age, female sex, more severe acute infection, more than five symptoms just in the first week of acute infection, immunosuppressive conditions, underlying health conditions (hypertension, obesity, psychiatric condition, etc.), and only partial or no vaccination [261,262,263,264,265,266].

As we have already discussed in this review article, long COVID could be induced by different mechanisms, including viral persistence that leads to chronic activation of the immune system and reactivation of latent viruses, the release of superantigens that over-activate the immune system, alteration in the gut microbiota, and damage to the gut and blood–brain barriers which collectively may result in autoimmunity and polyautoimmunity, including neuroautoimmunity [43,44,93,95,213,267,268,269,270,271,272,273,274,275,276].

In relation to the chronic activation of the immune system that is associated with the five hypotheses mentioned above, COVID-19 patients have shown long-term alterations for up to six months following their discharge in their primary B- and T-cell functions as well as in the vast majority of their CD4+ and CD8+ T-cell subsets, which are critical for the control of intracellular and extracellular pathogenic infections [277]. This long COVID-associated hyperactivation of Th17 and Tfh cells, changes in their subsets, the imbalance between regulatory and pro-inflammatory T-cell subsets in general, unregulated B-cell activation and antibody production increase the possibilities of autoimmune inflammation and autoimmune-related manifestations [124,127,251].

Based on our review of the literature, in both past and recent studies, we have found clues to these mechanisms that might drive this long COVID, with the goal of identifying host or virus factors that can be intervened upon to prevent or reverse this condition. A better understanding of these immunological mechanisms in patients with severe long COVID holds great promise for designing treatment strategies to minimize viral persistence, control the reactivation of latent viruses, and to modulate a dysregulated immune system and host microbiota, which together are involved in the virus-induced inflammation and autoimmunity that are observed in patients with long COVID.

## Figures and Tables

**Table 1:** Peptide sharing between human HSPs and SARS-CoV-2 proteins.

Shared Peptide	SARS-CoV-2 Protein (UniProt ID)	Human Chaperone (UniProt ID)	Putative Epitope
**EIPKEE**	Replicase polyprotein 1ab [P0DTD1]	Heat shock 60 kDa protein [P10809]	A**EIPKEE**VKPFITESKPSVEQRKQDDKK
**LPYPDP**	Replicase polyprotein 1ab [P0DTD1]	Heat shock 70 kDa protein [P34932]	TLEAYYSSPQD**LPYPDP**AIA
**KDKKKK**	Nucleocapsid phospho-protein [P0DTD1]	Heat shock protein HSP 90-beta [P08238]	KTFPPTEP**KDKKKK**ADETQALPQRQKKQQ

## Data Availability

Not applicable.
